# Structure, Properties, and Biomedical Activity of Natural Sweeteners Steviosides: An Update

**DOI:** 10.1002/fsn3.70002

**Published:** 2025-02-02

**Authors:** Aoyi Wang, Huiqin Hu, Yadan Yuan, Shiran Mei, Guoxue Zhu, Qiaoyan Yue, Yanliang Zhang, Shujun Jiang

**Affiliations:** ^1^ Nanjing Hospital of Chinese Medicine Affiliated to Nanjing University of Chinese Medicine Nanjing China; ^2^ Nanjing Research Center for Infectious Diseases of Integrated Traditional Chinese and Western Medicine Nanjing China

**Keywords:** biomedical activity, safety, stevioside

## Abstract

Stevioside is a natural sweetener with the characteristics of low calorie and high sweetness. It comprises a diverse range of monomers that play crucial roles in numerous biological processes. Due to these attributes, it has gained widespread application in agriculture, food, and pharmaceutical industries. As a substitute for sugar, stevioside also shows good pharmacological activities on glucose metabolism, bodyweight keeping, blood pressure maintenance, and shows anti‐inflammatory, anti‐oxidation, anti‐tumor, antibacterial, and immune regulation activities. This review summarized the update on the food safety, sweet structure–activity relationship, pharmacological activity of stevia glycosides recently, and discussed the limitations of its application in food and medicine.

## Graphical abstract

Stevioside is a natural sweetener with the characteristics of low calorie and high sweetness. It consists of a wide variety of monomers involved in several biological processes. It has been widely used in the food and pharmaceutical industries. As a substitute for sugar, stevioside also shows good pharmacological activities. On glucose metabolism, bodyweight keeping, blood pressure maintaining, and anti‐inflammatory, anti‐oxidation, anti‐tumor, antibacterial, and immune regulation activities. This review summarized the update on the food safety, sweet structure–activity relationship, pharmacological activity of stevia glycosides recently, and discussed the limitations of its application in food and medicine.

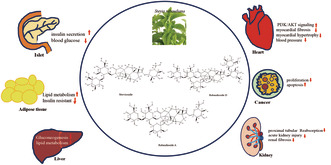

## 1 Introduction

Stevia, also known as sweet grass and sweet tea, is native to countries such as Paraguay and Brazil. The sweetening substance of stevia as a sweetener and sugar substitute is domesticated from the cultivated stevia ( *Stevia rebaudiana* ). The sweetness of stevia mainly consists of stevia glycoside and rebaudioside, which is 300 times sweeter than table sugar, and has the typical feature of good thermal stability and high acid–base stability. Stevia products are favored by consumers around the world for their pure natural, zero‐calorie, non‐fermentable, acid–alkali stability, no browning reaction, no fat and carbohydrate, anti‐dental caries, and generally recognized as safe (GRAS) (Ribeiro et al. 2021).

Stevia contains many different sweet ingredients, and 9 of these components have been fully investigated for medical toxicity, including (1) stevioside (STV); (2) steviolbioside; (3) rebaudioside A (Reb A); (4) rebaudioside B (Reb B); (5) rebaudioside C (Reb C); (6) rebaudioside D (Reb D); (7) rebaudioside E (Reb E); (8) rebaudioside F (Reb F); and (9) dulcoside A (DA). Among these glycosides, STV, Reb A, RC, and DA are four kinds with high content and economic value, while STV and Reb A account for more than 80% of the total glycosides, especially Reb A, is particularly popular owing to its sweetness and taste (Ribeiro et al. 2021). Recently, a total of 91 diterpene glycosides including steviol glycosides were identified, and 16 steviol glycosides were found with novel acetylglycosylation patterns through energy‐resolved (ER) untargeted liquid chromatography–mass spectrometry/mass spectrometry (LC–MS/MS) metabolomics analysis (Zhang, Li, et al. 2024; Zhang, Chen, et al. 2024). More research is needed to confirm whether these newly discovered STVs could be a better sweetener.

**FIGURE 1:**
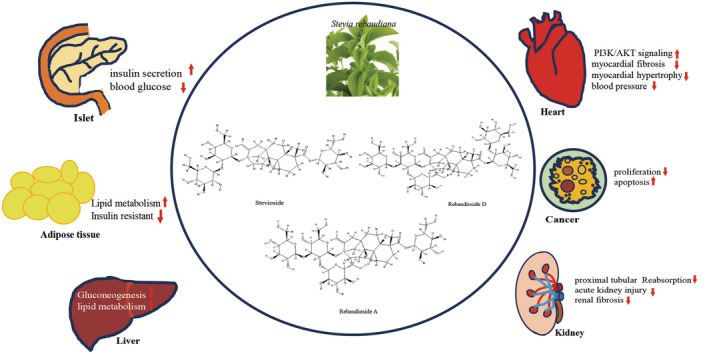
Biomedical activity of STV.

The special biomedical activities of stevia have recently received widespread attention (Figure 1), including in controlling and treating a variety of metabolic diseases especially obesity, diabetes, metabolic syndrome, and so on (Li, Wang, et al. 2021; Li, Zhu, et al. 2021; Carrera‐Lanestosa, Moguel‐Ordóñez, and Segura‐Campos 2017). Although numerous food regulatory and safety authorities globally have confirmed the safety of stevia, a lack of adequate education regarding its safety and benefits, coupled with ongoing concerns about the safety of low‐calorie natural sweeteners in general, discourages health professionals and consumers from endorsing or utilizing stevia. Recently, several lines of evidence suggest that stevia ingredients may be the new approaches to treating cardiovascular disease (CVD) and tumors (Olas 2022; Ilias, Ismail, et al. 2021; Ilias, Hamzah, et al. 2021; Ferrazzano et al. 2015). In this review, we have conducted a comprehensive survey of the literature to compile the findings from relevant studies and offer a concise summary that not only aids in consolidating current understanding on this topic but also identifies weaknesses in study design and suggests potential solutions to overcome them. The data examined in this article offer valuable insights into STV as a potential means for human disease prevention and treatment, especially within the framework of a healthy diet. Furthermore, STV proved to be an excellent additive for food, medical products, and feed in daily life.

This diagram visually depicts the complex biochemical pathways and interactions between STV derived from *S. rebaudiana* and various animal physiological systems. It emphasizes their potential roles in regulating blood glucose levels, blood pressure, lipid metabolism, and organ function, as well as their involvement in disease processes such as cancer and myocardial fibrosis. STV can safeguard islet function by stimulating insulin secretion and reducing blood glucose levels. Lipid metabolism enhancement helps restore insulin resistance. STV's hepatoprotective function is attributed to its inhibitory effect on gluconeogenesis and upregulation of lipid metabolism. Furthermore, STV alleviates both myocardial and kidney fibrosis. Its anti‐cancer effects stem from inhibiting proliferation and improving apoptosis. ↑The upward red arrow represents an activation effect and ↓The downward red arrow indicates a downregulation or inhibitory effect.

## 2 Structure and Property of STV

### 2.1 Physicochemical Properties of Reb A

**FIGURE 2:**
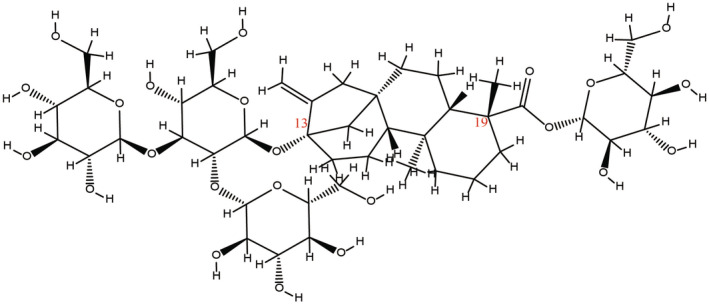
Chemical structure of Reb A.

Rebaudioside A (Reb A), also known as stevia bisoside A, is a natural sweetener extracted from the leaves of *S. rebaudiana Bertoni*. Among the steviol glycosides present in stevia leaves, Reb A constitutes a range of 1.5%–5.0%. Reb A is a tetracyclic diterpenoid glycoside containing 20 carbon atoms formed by an aglycone steviol, also known as a glycosyl linking one glucosyl at the C‐19 position and three glucosyls at the C‐13 position. The molecular structure of Reb A is shown in Figure 2. The attachment of one glucosyl group at the C‐19 position and varying numbers of glucosyl and rhamnosyl groups at the C‐13 position results in different levels of sweetness and taste profiles for Reb A (Jung et al. 2021). The sophorose group at the C‐13 position serves as the primary functional group for sweetness, while the ester group at the C‐19 position of the aglycone acts as a taste modifier. The connection of several glucosyl groups at the C‐13 position is necessary to achieve high sweetness intensity.

Reb A features a variable number of glucosyl and rhamnosyl groups attached at the C‐13 position, while a single glucosyl group is attached at the C‐19 position. The diversity in glycosyl groups linked to these two specific positions constitutes the primary factor contributing to the variations in sweetness and taste of Reb A.

The sweetness of sweeteners is directly correlated with sucrose equivalent sweetness (SE) concentration, solution pH, taste temperature, and other factors. Therefore, to describe the relative sweetness of Reb A, the solution system must be defined. Studies have shown that the relative sweetness of Reb A decreases from 435 times to 238 times when the isosaccharose concentration is increased from 2% to 10%, with a sweetness of 321 times at a 6% isosaccharose concentration (Wang et al. 2023). In contrast, the relative sweetness of aspartame was only 180 times at a 6% sweet sucrose concentration, significantly lower than that of Reb A. Although there have been disputes about the flavor evaluation of Reb A, most researchers agree that the sweetness of Reb A is generally closer to sucrose than other components of stevia, with no obvious sour, minty, metallic, and other bad aftertaste. The bitter and licorice flavors of Reb A are very low and can be ignored at low concentrations (SE ≤ 6); while its bitter and licorice taste will obviously affect the overall taste with the high amount of use (SE ≥ 6) (Yang, Liu, and Zhu 2017; Yadav and Guleria 2012).

### 2.2 Structure and Properties of Reb D and Reb M

**FIGURE 3:**
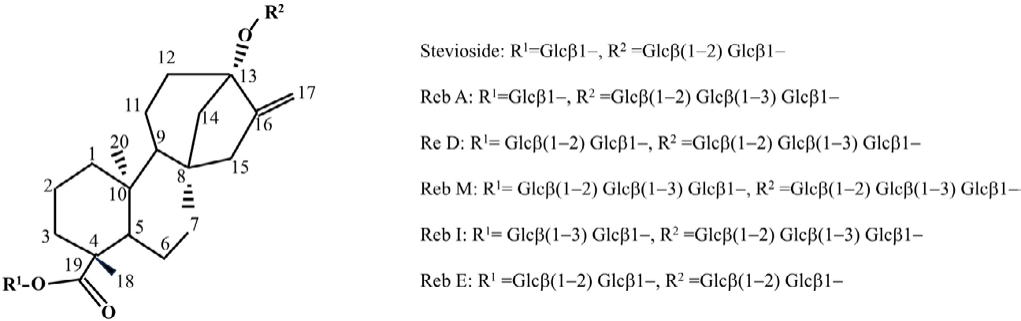
Chemical structure of major stevia glycosides.

Stevia glycosides are a class of glycosides formed by linking different numbers and types of sugar molecules through 1, 2‐, 1, 3‐, 1, 4‐, and 1, 6‐α or β glycosidic bonds at the C‐13 and C‐19 positions of the terpene aglycones of the core skeleton kaurene (Figure 3) (Ceunen and Geuns 2013).

**FIGURE 4:**
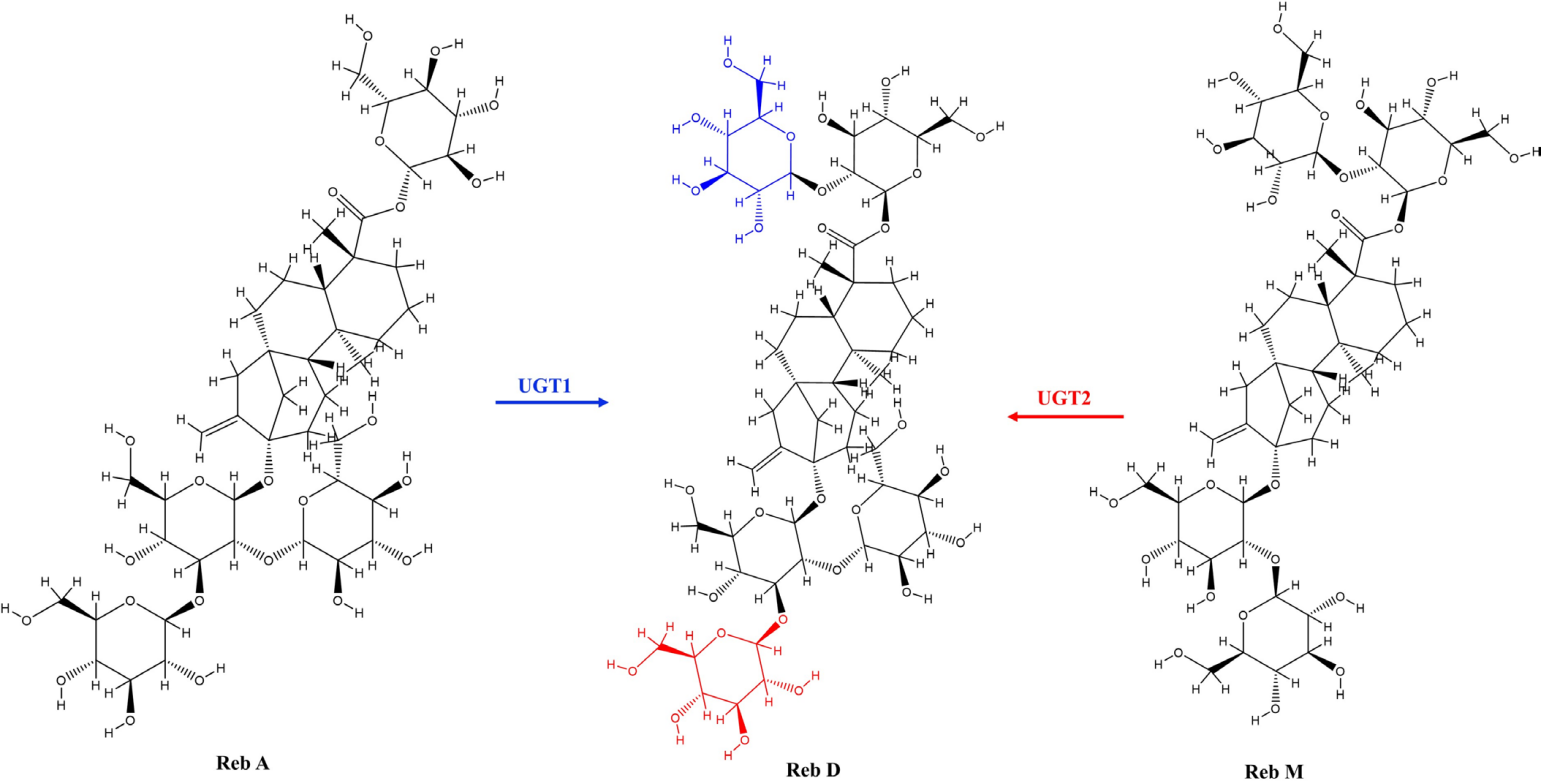
Glycosyltransferase catalyzes the synthesis of Reb D.

At present, more than 60 kinds of stevia glycosides have been isolated from Stevia. The diversity of their structures also leads to differences in their properties, such as sweetness, bitterness, and solubility. In 1977, the chemical structure of Reb D was first reported (Figure 3), the molecular formula is C_50_H_80_O_28_, and Reb D is formed by linking 2 and 3 glucosyl groups at the C‐19 and C‐13 positions of steviol (Sakamoto, Yamasak, and Tanka 1977). In 2014, the chemical structure of Rebaudioside M (Reb M) was first reported (Figure 3), with the molecular formula C_56_H_90_O_33_. Reb M is based on the structure of Reb D and is formed by linking a glucosyl group at the C‐19 position through β‐1, 3 glycosidic bonds (Prakash, Markosyan, and Bunders 2014). The content of Reb D and Reb M in stevia is relatively low, and the mass fraction is about 0.4%–0.5% (Chen et al. 2018). Therefore, the preparation by traditional plant extraction methods is not only costly but also difficult to meet the market demand. Reb D can be synthesized by glycosylation of Reb A/Reb E using uridine di‐phosphate glucose (UDPG)‐dependent glycosyltransferase (UGTs), which is further used to synthesize Reb M (Figure 4).

Compared with stevia and Reb A, Reb D, and Reb M have higher sweetness (about 250–350 times that of sucrose) and less back‐bitterness, which make them a new generation of sweeteners (Prakash, Markosyan, and Bunders 2014). In the human taste system, the perception of STV sweetness and bitte‐ness is mediated by the G protein‐coupled receptor protein of taste receptor cells in the taste buds. The heterozygous sweetness receptor proteins hTAS1R2 and hTAS1R3 mediated the sweetness of various chemical compounds including monosaccharides, disaccharides, sweet proteins, stevia glycosides, and synthetic non‐nutritional sweeteners (Hellfritsch et al. 2012).

The differences in the structure of stevia glycosides result in their sweetness, bitterness, and aftertaste. In a time‐intensity dynamic sensory model study of the sweet and bitter taste of six stevia glycosides, it was found that Reb D and Reb M had faster sweetness and shorter aftertaste with almost no aftertaste taste compared with sweet tea glycosides, stevia, Reb A, and Reb C. Conversely, sweet tea glycosides and stevia demonstrated pronounced bitterness and enduring aftertastes (Tian, Zhong, and Xia 2022). In addition, the study also found that the less glucosyl group of stevia glycoside C‐19, the shorter the sweetness stimulation time and the longer the perception time of bitterness. The more glucosyl groups at C‐13, the stronger and faster the sweetness was. The reason for this phenomenon is that as the substituents in the C‐19 position of stevia glycoside become larger and more numerous, they increase the desorption rate, leading to a quicker decay of sweetness. While compounds with fewer sugar groups, such as stevia itself, exhibit a lower desorption rate, resulting in a lingering bitter aftertaste (Tian, Zhong, and Xia 2022).

## 3 Pharmacological Function

**FIGURE 5:**
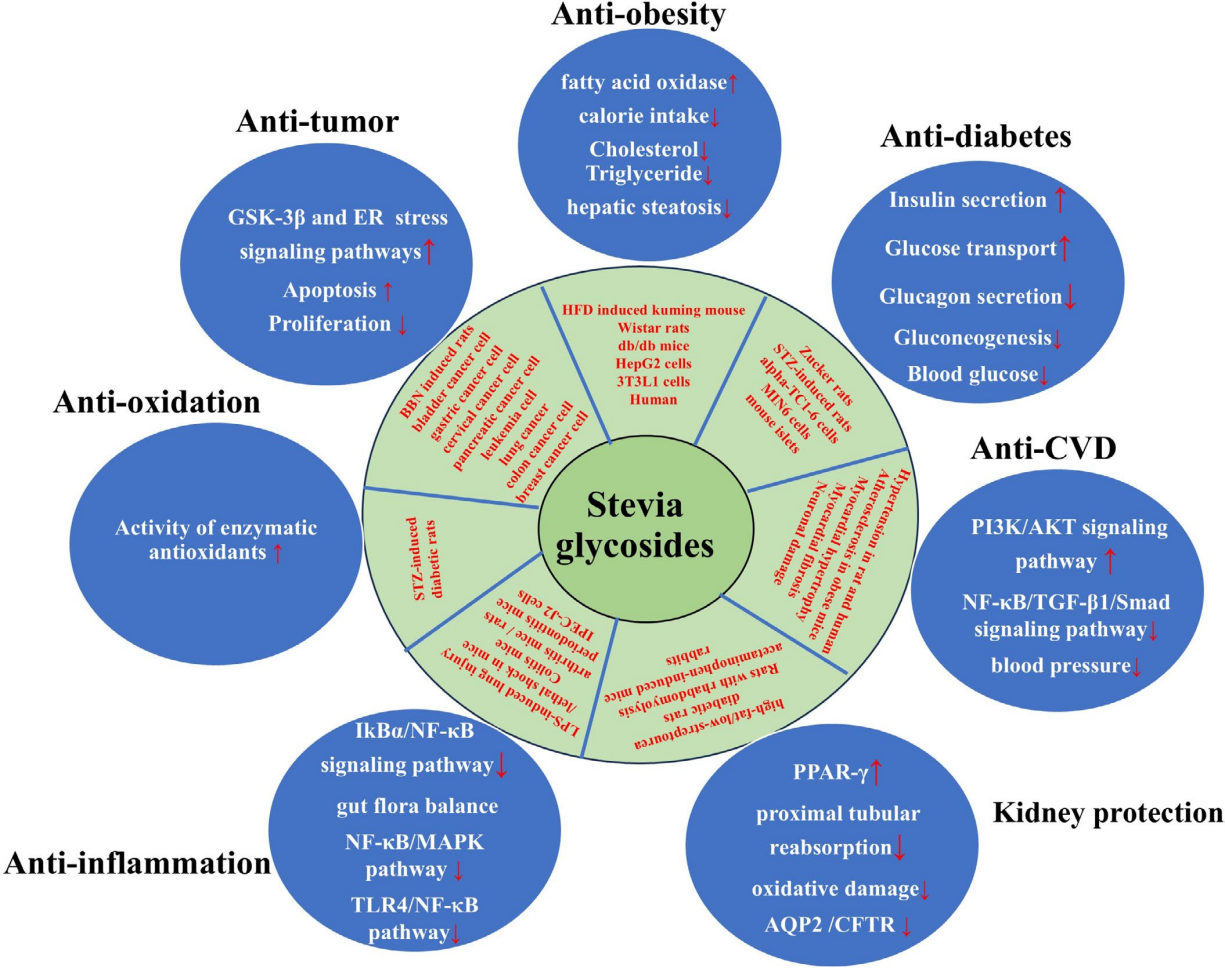
Pharmacological activity of stevia glycosides. AQP2, Aquaporin 2; CFTR, cystic fibrosis transmembrane conductance regulator; ER, endoplasmic reticulum; GSK‐3β, glycogen synthase kinase 3 β; MAPK, mitogen‐activated protein kinase; NF‐κB, nuclear factor kappa‐B; PI3K/AKT, phospha‐tidylinositol‐3‐kinase/protein kinase B; PPAR‐γ, peroxisome proliferator‐activated receptor γ; TGF‐β, transforming growth factor‐β; TLR4, Toll‐like receptor 4; ↑The upward red arrow represents an activation effect; ↓The downward red arrow indicates downregulation or inhibitory effect.

Stevia glycosides, which possess no teratogenic, carcinogenic, or mutagenic effects, exhibited good effects on anti‐diabetes, anti‐oxidation, high blood pressure, prevent dental caries, obesity, antivirus, and antitumor biological activity (Figure 5).

### 3.1 Regulation of Glycolipid Metabolism

#### 3.1.1 Promote Insulin Secretion

Several studies have shown that STV and Reb A can promote insulin secretion by pancreatic islet β cells, improve glucose metabolism, and inhibit the decomposition of fatty acids and bile acids, which contribute to weight loss (Barriocanal et al. 2008; Gregersen et al. 2004; Reynolds 4th et al. 2017). They are currently recognized as insulin stimulation and hypoglycemic drugs (Semwal et al. 2021). STV acts directly on beta cells, inhibiting glucagon secretion by alpha cells of the pancreas without altering ATP‐sensitive K (+)‐channels activity and cAMP (Jeppesen et al. 2000), enhancing insulin secretion and lowering blood sugar levels (Shibata et al. 1995; Han, Jio, et al. 2023; Han, Park, and Lee 2023). Some studies have also found that low concentrations of STV can significantly affect glucose transport in skeletal muscles (Lailerd et al. 2004). In addition, STV can attenuate gluconeogenesis in the rat liver, reduce the gene and protein expression of phosphoenolpyruvate carboxykinase (PEPCK), inhibit the activity of α cells and β cells with long‐term accumulation of fatty acids in the pancreas (Chen et al. 2005), promote the expression of genes related to fatty acid metabolism, and improve fatty acid homeostasis (Hong et al. 2006).

It was also clearly verified that STV can enhance insulin secretion by pancreatic islet beta cells in diabetic rats (Shivanna et al. 2013). Previous study has shown that Reb A (10^−16^–10^−6^ mM) promoted islet secretion under high glucose (16.7 mM glucose) stimulation and in the presence of extracellular calcium ions, in a concentration‐dependent manner. Stimulation of insulin release occurred at concentrations of 10^−14^ mol/L, with maximum insulin response obtained at 10^−10^ mol/L (Abudula et al. 2004). Reb A stimulates insulin secretion within the range of 3.3–16.7 mmol/L, specifically enhancing insulin secretion only when glucose levels exceed 6.6 mmol/L. The action of Reb A is heavily dependent on the presence of extracellular Ca^2+^, in the absence of extracellular Ca^2+^, the insulin‐stimulating effect of Reb A in response to high glucose levels diminishes. Reb A has an insulinotropic effect and may be a potential treatment for type 2 diabetes mellitus (Abudula et al. 2004).

#### 3.1.2 Lower Blood Glucose

Multiple lines of evidence have demonstrated that STV can significantly reduce the blood glucose level in different diabetic rat models (Ilić et al. 2017; Aswar et al. 2019; Kurek et al. 2020; Kurek et al. 2022). The hypoglycemic effect of STV is largely attributed to its ability to promote insulin secretion. Studies have shown that 1 mg STV can reduce postprandial blood glucose by 18% and increase the insulin‐to‐glucose ratio in serum by 40% (Gupta et al. 2016). Consistent with this, an in vitro study has provided evidence that STV reduced glucose dependence, balanced energy intake, improved glucose metabolism regulation, and promoted pancreatic recovery by stimulating insulin secretion in MIN6 cells (Abudula et al. 2008). A meta‐analysis of randomized controlled trials (RCTs) demonstrated that steviol glycosides (SGs) significantly improve glucose metabolism in adult participants aged 50 years or younger, who were overweight or obese, and did not have diabetes mellitus (DM) or hypertension at baseline, when compared to the control group (Bai et al. 2024). Therefore, utilizing low‐calorie, naturally sweet‐tasting, and high‐quality STV and Reb A as alternatives to sugar represents a novel strategy for the prevention and management of diabetes.

#### 3.1.3 Protect of Islet Function

Transient receptor potential cation channel, subfamily M, member 5 (TRPM5) is a Ca^2+^‐activated cation channel expressed in type II taste receptor cells and pancreatic β cells. It has been reported that STV, Reb A, and its glycosyl steviol can target TRPM5 to enhance the function of pancreatic islets and promote insulin secretion induced by high fat in mice (Philippaert et al. 2017). Another study also proved that stevia leaf extract has a protective effect on beta cells exposed to lipotoxicity (Bugliani et al. 2022). Consistent with these data, there is ample evidence that stevia glycosides can reduce blood sugar levels and alleviate islet cell damage in diabetic rats (Kurek, Mikołajczyk‐Stecyna, and Krejpcio 2023).

#### 3.1.4 Lose Weight and Lipid

A previous study found that rats with stevia diet show significantly lower body weight, total cholesterol, triglycerides, and low‐density lipoprotein (LDL), while HDL increased compared to rats with sucrose (Abo Elnaga et al. 2016) 0.150 mg/kg of stevia leaf ethanol extract significantly reduced blood glucose, total cholesterol, and triglyceride level in diabetic rats induced by tetraoxane (Hossain et al. 2011). Similarly, the glucose and lipid‐lowering effects of stevia extract (STE) were verified in high‐sugar and high‐fat diet Kunming mouse. Stevia glycosides might also regulate lipid metabolism, maintain oxidative stress balance, and inhibit lipid accumulation in the liver (Chen et al. 2023; Saadi et al. 2024).

STV and Reb A (500 or 2500 mg/kg for 5 weeks) modulated lipid metabolism‐related genes to alleviate lipid metabolism abnormalities in streptozotocin (STZ) and high‐fat‐induced diabetic Wistar rats (Morissette et al. 2024). It also proved that Reb A accelerated the absorption of LDL cholesterol by inhibiting the expression of 3‐hydroxy‐3‐methylglutaryl‐CoA reductase (HMGCR) in HepG2 cells (Ilias, Ismail, et al. 2021). The study of hypocaloric sweeteners *S. rebaudiana* Bertoni and STV on hepatic steatosis and hepatocyte autophagy showed that S and SS increased the levels of fatty acid oxidase, peroxisome proliferator‐activated receptor α (PPAR) and microtubule‐associated protein light chain 3b, and decreased the levels of lock sequestosome 1 (p62) in the liver of *db/db* mice, and knockout of PPAR blocked STV‐mediated lipid phagocytosis of HepG2 cells (Park, Sharma, et al. 2022). It has also been shown that stevia glycosides can promote lipid metabolism by enhancing b‐oxidation and activating adenosine monophosphate‐activated protein kinase (AMPK) signaling pathway both in vitro and in vivo (Park, Baek, et al. 2022). Clinical studies have also confirmed that replacing sugar cubes with natural sugar stevia could help overweight Indians reduce calorie intake and weight loss (Aswathiah et al. 2022; Raghavan et al. 2023). The latest study confirmed that C57BL/6 male mice were administered steviol glycoside, specifically Reb M at a concentration of 536.25 mg/L in their drinking water for a duration of 20 weeks, they exhibited enhanced insulin sensitivity and reduced weight gain (Rathaus et al. 2024). In summary, these data suggested that STV is a potential candidate for the treatment of hepatic lipid metabolism disorders, primarily due to its capacity to stimulate lipid oxidation.

In summary, the mechanisms by which steviol glycoside influences glycolipid metabolism involve the enhancement of insulin secretion, the amelioration of insulin resistance, the maintenance of glucose homeostasis, and the promotion of lipolysis. These effects are mediated through the potentiation of Ca^2+^‐dependent transient receptor potential cation channel subfamily melastatin member 5 (TRPM5) activity, the facilitation of adenosine monophosphate (AMP)‐activated protein kinase (AMPK) phosphorylation, the modulation of lipid autophagy, and the upregulation of transcript expression for Carnitine palmitoyltransferase 1 (CPT1), peroxisome proliferator‐activated receptor γ (PPARγ), stearoyl‐CoA desaturase (SCD), low‐density lipoprotein receptor (LDLR), and acetyl‐CoA acetyltransferase 2 (ACAT2). Additionally, steviol glycoside downregulates the expression of phosphoenolpyruvate carboxykinase (PEPCK), lipopolysaccharide‐binding protein (LBP), and 3‐hydroxy‐3‐methylglutaryl coenzyme A reductase (HMGCR), among others (Table 1).

**TABLE 1:** The regulation of stevia glycosides in glycolipid metabolism.

Species	Models	Types	Intervention	Doses	Effects and related mechanisms	References
Cell	Islet from female NMR1 mice β‐cell line INS‐1	In vitro		1 nm–1 mm stevioside/steviol	Stimulate insulin secretion via a direct action on b‐cell without impacting the plasma membrane K + adenosine triphosphate (K ~ Tp)‐Sensitive channel activity, and cyclic adenosine monophosphate (cAMP) levels in islets(	Jeppesen et al. (2000)
	Islet from female NMR1 mice	In vitro		10^−16^ to 10^−6^ M rebaudioside A	promoted insulin secretion on calcium dependent	Abudula et al. (2004)
	Islet from female NMR1 mice insulinoma MIN6 cells	In vitro		10^−13^ to 10^−7^ M rebaudioside A	Stimulated insulin secretion through inhibition of ATP‐sensitive K^+^‐channels(	Abudula et al. (2008)
	α‐tumor cell 1 clone 6 (α‐TC1‐6) cells	In vitro		10^−8^ to 10^−6^ M stevioside	Enhanced expression of genes involved in fatty acid oxidation (CPT 1), fatty acid disposal (PPARγ), and fatty acid conversion (SCD)	Hong et al. (2006)
	INS‐1E beta cells and human islets	In vitro		200 μg/mL of a stevia extract	Alleviated endoplasmic reticulum stress and mitochondrial dysfunction induced by palmitate	Bugliani et al. (2022)
	HepG2 cells	In vitro		0–10 μm Reb A	Ameliorated cholesterol accumulation by downregulated HMGCR, upregulated LDLR, and ACAT2	Ilias, Ismail, et al. (2021)
	HepG2 cells	In vitro		0, 12.5, 25, 50, and 100 μM stevioside	Augmented autophagy by increased LC3B‐II/LC3B‐I levels and reduced SQSTM1 (p62) levels in steatosis‐induced hepatocytes	Park, Sharma, et al. (2022)
	3T3‐L1 pre‐adipocytes	In vitro		0, 25, 50, and 100 μM stevioside	Downregulated lipid accumulation	Park, Baek, et al. (2022)
Rat	Streptozotocin induced Wistar male rat	In vivo	Per Os	4% stevia leaf powder/polyphenols/fiber in the diet	Reduced blood glucose, ALT, AST, and oxidative stress; increased insulin level; alleviated liver and kidney injury	Shivanna et al. (2013)
	Streptozotocin‐induced Wistar male rat	In vivo	Per Os	0.5, 1, or 5 mg/kg stevioside	Raised insulin secretion and decreased PEPCK	Chen et al. (2005)
	Streptozotocin‐induced Wistar rats	In vivo	Per Os	500 or 2500 mg/kg steviol glycosides (stevioside or rebaudioside A)	Downregulated hepatic Fasn gene, increased mRNA level of Glut4 in muscle, upregulated expression of Cebpa in the liver	Kurek, Mikołajczyk‐Stecyna, and Krejpcio (2023)
	Tetraoxane‐induced Wistar rats	In vivo	Per Os	150 mg/kg of stevia leaf ethanol extract	Reduced blood glucose, total cholesterol, and triglyceride level	
Mouse	C57Bl6/J mice, Trpm5 knock out mice, Tas1r2 and Tas1r3 knock out mice	In vivo	Per Os	25 mg stevioside per kg in a 0.1% solution in water	Potentiated glucose‐induced insulin secretion and glucose tolerance via enhancing Ca^2+^‐dependent TRPM5 activity	Philippaert et al. (2017)
	High‐fat diet male mice (NMRI Haan strain)	In vivo	Per Os	20 mg/kg stevioside	Alleviated diabetes treated with low‐dose stevioside	Ilić et al. (2017)
	High‐sugar and high‐fat diet Kunming mouse	In vivo	Per Os	50 mg/kg stevia leaf ethanol extract	Promoted lipid metabolism, reduced serum lipids (triglycerides, cholesterol, low‐density lipoprotein, and free fatty acids), increased serum levels of antioxidant enzymes (SOD and GSH‐Px); increased ratio of Firmicutes/Firmicutes	Hossain et al. (2011)
	High‐fat/high‐sucrose (HFHS) male C57BL/6 J mice	In vivo	Per Os	50 mg/kg/day of RebA or RebD	Increased glycolipid metabolism by reduced LBP	Morissette et al. (2024)
	High‐fat diet male C57Bl6/J mice	In vivo	p.o	Steviol glycoside (Reb M, 536.25 mg/L)	Improved insulin sensitivity and attenuates weight gain in obese mice	Rathaus et al. (2024)
	*db/m+* and *db/db* mice	In vivo	Per Os	200 or 500 mg/kg stevioside 40 mg/kg stevia extract	Activated insulin signaling by promoting the protein expression of IRS, Akt, and Glut 4	Han, Jio, et al. (2023, Han, Park, and Lee (2023)
	*db/m +* and *db/db* mice	In vivo	Per Os	200 or 500 mg/kg stevia leaf extract; or 40 mg/kg stevioside	Attenuated hepatic steatosis via increased levels of fatty acid oxidase, PPARα, and LC3 B but decreased that of p62	Park, Sharma, et al. (2022)
	*db/m +* and *db/db* mice	In vivo	Per Os	40 mg/kg stevioside	Promoted β‐oxidation in adipocytes by increasing the phosphorylation of AMPK and ACC	Park, Baek, et al. (2022)
Human	Normal Indian	In vivo	Per Os	2.5 g sugar blend (a novel combination of sugar and stevia)	Promoted weight loss and decreased serum lipid	Aswathiah et al. (2022)
	Overweight prediabetic Indian	In vivo	Per Os	0–4 mg/kg stevia‐based powder formulation and pellet contained 2.19% w/w and 20.51% w/w of steviol glycosides	Enhanced weight loss and diminishes waist circumference among overweight individuals with normal blood glucose levels as well as those in the prediabetic stage	Raghavan et al. (2023)
	Diabetic patient	in vivo	Per Os	1 mg/kg stevioside	Blood glucose levels decreased by 18%, and the ratio of insulin to glucose in serum increased by 40%	Gupta et al. (2016)

Abbreviations: ACAT2, acetyl‐CoA acetyltransferase 2; ACC, acetyl‐CoA carboxylase; Akt, serine/threonine kinase B; ALT, alanine aminotransferase; AMPK, Adenosine 5′‐monophosphate (AMP)‐activated protein kinase; AST, aspartate aminotransferase; ATP, adenosine triphosphate; cAMP, cyclic adenosine monophosphate; Cebpa, CCAAT/enhancer binding protein α; CPT 1, carnitine palmitoyltransferase 1; Glut4, glucose transporter type 4; GSH‐Px, glutathione peroxidase; HMGB1, high mobility group protein B1; IRS, insulin receptor substrate; LBP, lipopolysaccharide‐binding protein; LC3B‐II/LC3B‐I, microtubule‐associated protein light chain 3 B; LDLR, low‐density lipoprotein receptor; PEPCK, phosphoenolpyruvate carboxykinase; PPARα, peroxisome proliferator‐activated receptor alpha; PPARγ, peroxisome proliferator‐activated receptorγ; RAC‐alpha serine/threonine‐protein kinase; SCD, stearoyl‐CoA desaturase; SOD, superoxide dismutase; SQSTM1 (p62), sequestosome 1; TRPM5, transient receptor potential cation channel, subfamily M, member 5.

### 3.2 Anti‐Inflammatory Effect

Several lines of evidence suggest that STV and its derivatives (including steviol, isosteviol, and sodium isosteviol) have good anti‐inflammatory effects (Boonkaewwan and Burodom 2013; Hanet et al. 2019; Alavala et al. 2020; Wei et al. 2021; Zhang et al. 2022; Xu, Liu, and Zhang 2023a; Xu et al. 2023b). Lipopolysaccharides (LPS), as an endotoxin, are a unique component of the cell wall of Gram‐negative bacteria. It can activate the host's immune response, produce inflammatory cytokines, cause excessive activation of the immune system, and induce sepsis and multiple organ dysfunction. Previous studies have found that STV and steviol might affect cytokine gene expression through the inhibitor of nuclear factor‐κB α/nuclear factor κB (IkBα/NF‐κB) signaling pathway, thereby attenuating the production of pro‐inflammatory cytokines induced by lipopolysaccharide (Xu, Liu, and Zhang 2023a). It has been proved that stevia glycosides could protect mice from LPS‐induced lethal shock by AMPK activation and macrophage inflammation inhibition (Wei et al. 2021). Comparing the effects of three common sweeteners on dextran sodium sulfate‐induced colitis in mice, sodium saccharin had the most significant improvement effect on colitis, followed by stevia glycoside and sucralose (Boonkaewwan and Burodom 2013). Oral administration of sweetness attenuated colitis symptoms through altered gut flora, protected gut barrier, remodeled TH17/Treg balance, and reduced inflammation in colitis mice (Zhang et al. 2022). In addition, STV could also improve the antioxidant capacity and intestinal barrier function of diquat‐induced IPEC‐J2 cells by regulating the NF‐κB/MAPK pathway, while reducing inflammation and apoptosis (Xu et al. 2023b). It has also been found that maternal addition of stevia glycosides might ameliorate intestinal mucosal damage and regulate intestinal flora homeostasis in chickens stimulated by lipopolysaccharide (Jiang et al. 2021). The above studies confirmed that stevia glycoside has a good effect on inhibiting intestinal inflammation.

Osteoarthritis (OA) is a common and progressive joint chronic disease in which stevia glycosides block interleukin‐1β‌‌ (IL‐1β) from eliciting an inflammatory response in mouse chondrocytes and block cartilage degradation in vivo through integrin α_V_β3 (Wan et al. 2023). Integrin α_V_β3 is highly expressed in osteoclasts and participates in their differentiation and maturation. Activation of integrin α_V_β3 signaling in many articular cell types contributes to inflammation and joint destruction in OA (Wang, Onuma, et al. 2019). It has also been confirmed that STV could inhibit chondrocyte inflammation and apoptosis through NF‐κB and MAPK pathways in vivo, and alleviate osteoarthritis (Cai et al. 2023). STV show good anti‐inflammatory efficacy against Freund's complete adjuvant (FCA)‐induced adjuvant arthritis in rats (Wu et al. 2022). All the above studies suggested that stevioside might be used as a new treatment option for OA.

The steviol with a kauri‐type tetracyclic diterpene skeleton structure is the constituent aglycone of STV, which shows anti‐bacterial and anti‐inflammatory effects (Voloshina et al. 2021). Isosteviol, a derivative of steviol, exhibited a good anti‐inflammatory effect on different mouse models (Chen, Yi, et al. 2024; Chen, Li, et al. 2024; Qiao et al. 2022; Wang, Tan, et al. 2021; Tang et al. 2018; Yao et al. 2023). Isosteviol can reduce inflammation by promoting the increase of the proportion of M2/M1 macrophages and up‐regulating the expression of matrix metalloproteinase 9 (MMP9) in burn wound tissue during acute inflammation (Qiao et al. 2022). The reduction in the percentage of M1 macrophages with a concomitant increase in the proportion of promyogenic M2 macrophages elevated the MMP9 level. Sodium isosteviol (STV‐Na), a terpenoid extracted from STV, also exhibited anti‐inflammatory and anti‐oxidative stress properties under different stimulations (Mei et al. 2020; Wang et al. 2022; Wang, Huang, et al. 2021; Wang, Tan, et al. 2021). Previous data verified that STV‐Na attenuated lung histopathological changes in LPS‐induced lung injury in mice, reduced the infiltration and oxidative stress of inflammatory cells in lung tissue, and alleviated inflammatory responses in mouse lung tissue through the TLR4/NF‐kB pathway (Xu, Liu, and Zhang 2023a; Xu et al. 2023b). Consistent with this, a recent study has also confirmed the protective effect of isosteviol sodium on LPS‐induced multi‐organ damage by regulating glycerophospholipid metabolism and reducing macrophage inflammatory response (Wang, Huang, et al. 2021). Furthermore, sodium isosteviol has the potential to ameliorate high‐fat/high‐cholesterol‐induced kidney disorders, myocardial dysfunction, and nonalcoholic fatty liver disease (NAFLD) through the activation of the Sirtuin 1 (Sirt1)/AMPK signaling pathway and the suppression of inflammation (Mei et al. 2022a, 2022b, 2020). These data suggest that STV derivatives exhibit favorable anti‐inflammatory properties on various diseases as well.

Stevia glycosides have demonstrated their efficacy in inhibiting inflammation caused by various conditions, including bacterial infection (Han, Jiao, et al. 2023; Han, Park, and Lee 2023; Wang, Song, et al. 2014; Wang, Guo, et al. 2014), acute liver injury (Latha, Chaudhary, and Ray 2017), metabolic disorders (Wang et al. 2012; Sánchez‐Tapia et al. 2019), and obesity (Dandin et al. 2022). Recent animal experiments have further revealed that, when compared to a 10% glucose intake, a 0.1% stevia glycoside intake can significantly reduce alveolar bone resorption and periodontal tissue inflammation, while also regulating bacterial homeostasis in mice with periodontitis (Han, Jiao, et al. 2023; Han, Park, and Lee 2023). Additionally, it also has shown that high concentrations of stevia glycosides decreased the activity, biofilm formation, and virulence expression of *Porphyromonas gingivalis* , a Gram‐negative, obligate anaerobic bacterium that does not ferment sugars bacterium commonly associated with periodontal disease (Han, Jiao, et al. 2023; Han, Park, and Lee 2023). In conclusion, various studies have consistently confirmed the anti‐inflammatory effects of STV and its derivatives on different models. However, further research is required to elucidate the specific targets and mechanisms of action involved.

As illustrated in Table 2, STV and its derivatives exhibit good anti‐inflammatory activity against LPS, DSS, bacterial, and high‐fat‐induced inflammatory models. Notably, variations in the mode of drug administration had no impact on its anti‐inflammatory efficacy. Their primary mechanism of action involved inhibiting macrophage polarization and suppressing inflammation‐related signaling pathways such as NF‐κB, ERK, MAPK, and AMPK signaling pathways.

**TABLE 2:** Anti‐inflammatory activities of steviol glycoside.

Species	Model	Cell/animal	Inducer	Dosage	Phenotype/mechanism	Reference
Cell	Colonic inflammation	Caco‐2 cell	LPS	Stevioside (0.001–1 mmol/L) steviol (0.1–100 μmol/L)	Alleviated LPS‐induced inflammation via IκBα/NF‐κB signaling pathway	Boonkaewwan and Burodom (2013)
	Oxidative stress	IPEC‐J2 cells	Diquat	0, 50, 100, 250, and 500 μM stevioside	Raised antioxidant capacity and inhibited inflammation by interfering with the NF‐κB and MAPK signaling pathways	Xu et al. (2023b)
	Mastitis	Mouse mammary epithelial cells isolated from BALB/c mice	*Staphylococcus aureus* SA113 (ATCC35556)	300, 100, or 30 μg/mL stevioside	Inhibited inflammatory and apoptosis by modulating NF‐κB and MAPK signaling pathways, as well as apoptosis‐related factors	Wang, Song et al. (2014)
Mouse	Mastitis	BALB/c mice	*Staphylococcus aureus* SA113 (ATCC35556)	300, 100, and 33 mg/kg stevioside (p.o)	Suppressed inflammation by reducing the phosphorylation of Iκ‐Bα and p65 in the NF‐κB pathway and the phosphorylation of p38, ERK, and JNK in the MAPK pathway	Wang, Guo, et al. (2014)
	Lung injury	Male BALB/c	LPS	20 mg/kg sodium isosteinol (STV‐Na) (i.p. injection)	Reduced inflammatory response and oxidative stress via the TLR4/NF‐kB pathway	Xu, Liu, and Zhang (2023a)
	Multiple organ injury	Male BALB/c	LPS	5, 10, and 20 mg/kg Isosteviol sodium (STV‐Na) (i.p. injection)	Inhibited macrophage infiltration by upregulating the inflammatory response and lipid, nucleic acid, and amino acid metabolism	Wang, Huang, et al. (2021)
	Lethal shock	Female BALB/C mice and C57BL/6 mice	D‐GalN/LPS	10, 20, and 40 mg/kg stevioside (i.p. injection)	Inhibited LPS‐induced pro‐inflammatory factors (IL‐6, TNF‐α, IL‐1β, NO, COX2, and HMGB1), promoted anti‐inflammatory cytokines synthesis in macrophages, activated AMPK signaling in the periphery blood mononuclear cells	Wei et al. (2021)
	Inflammatory bowel diseases (IBDs)	Male BALB/c mice	DSS	10 mg/kg STV‐Na (i.p)	Suppressed the NF‐κB/p65 signaling pathway, decreased M1 macrophage polarization	Wang et al. (2022)
	Colitis	C57BL/6 mice	DSS	5 mg/kg stevioside in drinking water	Enhanced the expression of E‐cadherin via the miR‐15b/RECK/MMP‐9 axis to bolster the intestinal barrier integrity. Attenuated inflammation by inhibition of MMP‐9/AKT/NF‐κB pathway	Zhang et al. (2022)
	Colitis	Male C57BL/6J mice	DSS	10 and 15 mg/kg STV‐Na (i.p. injection)	Ameliorated intestinal inflammation through inhibition of macrophage infiltration and polarization	Wang, Tan, et al. (2021)
	Colitis	Male C57BL/6J mice	DSS	10 mg/kg isosteviol (i.p)	Ameliorated intestinal barrier injury by downregulating PDK1/AKT/NF‐κB signaling pathway	Yao et al. (2023)
	Osteoarthritis	Male C57BL/6J mice	Surgical destabilization of the meniscus	50 mg/kg stevioside (intra‐articular injection)	Downregulated inflammation by inactivating PI3K/Akt/NF‐κB and MAPK signaling pathways, prevented cartilage degradation through integrin αVβ3	Wan et al. (2023)
	Osteoarthritis	Male C57BL/6J mice	Surgical destabilization of the meniscus	100 mg/kg stevioside (p.o)	Prohibited the activation of MAPK and NF‐κB signaling pathways by targeting p65, ERK, p38, and JNK	Cai et al. (2023)
	Osteoarthritis	Male C57BL/6J mice	Surgical destabilization of the meniscus	50 mg/kg stevioside (p.o)	Ameliorated osteoarthritis through Nrf2/HO‐1/NF‐κB signaling pathway	Wu et al. (2022)
	Periodontitis	Male C57BL/6J mice	*Porphyromonas gingivalis*	0.1% stevioside in drinking water	Inhibited periodontitis by reducing the expression of inflammatory cytokines	Han, Jiao, et al. (2023), Han, Park, and Lee (2023)
	Insulin resistance	Male C57BL/6J mice	HFD	10 mg/kg stevioside (p.o)	Ameliorate insulin resistance by attenuating adipose tissue inflammation and inhibiting the NF‐κB pathway	Wang et al. (2012)
Rat	Adjuvant arthritis	Male SD rats	Freund's complete adjuvant (FCA)with 5 mg/mL of *Mycobacterium tuberculosis* H37Ra	62.5, 125 and 250 mg/kg stevioside (p.o)	Reduced the inflammation by inhibiting the pro‐inflammatory cytokines, and increased the anti‐oxidant levels	Alavala et al. (2020)
	Kidney dysfunction	Male SD rats	HFD	1, 10, and 20 mg/kg STV‐Na (p.o)	Attenuated inflammation, oxidative stress, and apoptosis by inhibition of the NF‐κB pathway	Mei et al. (2020)
	Myocardial dysfunction	Male SD rats	HFD	1, 10, and 20 mg/kg STV‐Na (p.o)	Alleviated inflammation and oxidative stress by the activation of the Sirt1/AMPK network and improved lipid metabolism	Mei et al. (2022a)
	Nonalcoholic fatty liver disease (NAFLD)	Male SD rats	HFD	1, 10, and 20 mg/kg STV‐Na (p.o)	Increased lipid metabolism by activating autophagy via the Sirt1/AMPK pathway	Mei et al. (2022b)
	Diabetic cardiomyopathy	Male Wistar rat	Streptozotocin	8 mg/kg STV‐Na (p.o)	Prevented diabetes‐inducted ERK and NF‐κB signal pathways without influence on blood glucose, plasma glycation end products AGEs, and insulin levels	Tang et al. (2018)
	Liver injury	Male Wistar rats	LPS	250 mg/kg stevioside or 250 mg/kg hydroalcoholic extract *Stevia rebaudiana* leaves (p.o)	Attenuated hepatic inflammatory response and oxidative stress via downregulating proinflammatory cytokines (TNF‐α, IL‐1β)	Latha, Chaudhary, and Ray (2017)
Hen	Intestinal mucosal damage	Jinmao yellow‐feathered breeder hens	LPS	250 mg/kg stevioside (p.o)	Attenuated intestinal mucosal damage and modulated gut microbiota in the chick offspring challenged with LPS	Jiang et al. (2021)
Zebrafish	Obesity	Wild‐type AB/AB strain zebrafish	Cysts overfeeding	1 and 5 mg/kg stevioside (p.o)	Improved glycolipid metabolism by reducing inflammatory and oxidative stress	Dandin et al. (2022)

Abbreviations: AGEs, plasma glycation end products; AMPK, adenosine 5′‐monophosphate (AMP)‐activated protein kinase; COX2, Cyclooxygenase‐2; DSS, dextran sodium sulfate; ERK, extracellular signal‐regulated kinase; HFD, High‐fat diet; HMGB1, high mobility group protein B1; HO‐1, Heme Oxygenase 1; IL‐1β, interleukin‐1β; IL‐6, interleukin‐6; IκBα, NF‐kappa‐B inhibitor alpha; JNK, c‐Jun N‐terminal kinase; LPS, lipopolysaccharide; MAPK, mitogen‐activated protein kinase; miR‐15b, microRNA‐15b; MMP‐9, MMP‐9, Matrixmetalloproteinase‐9; NF‐κB, nuclear factor kappa B; NF‐κB, nuclear factor κB; Nrf2, nuclear respiratory factor 1; NOS, nitric oxide synthase; PI3K/Akt/NF‐κB, phosphoinositide‐3‐kinase/Akt/nuclear factor‐kappa beta; RECK, reversion inducing cysteine‐rich protein with Kazal motifs; SD, Sprague Dawley; Sirt1, sirtuin3; TLR4, Toll‐like receptor 4; TNF‐α, tumor necrosis factor α.

### 3.3 Antioxidant Effect

Reports indicated that stevia glycosides could promote lipid oxidation. Yu Hui et al. reported that aqueous extract from *S. rebaudiana* stem waste exhibited significant antioxidant activity against fish oil oxidation fish oil (Yu et al. 2017). Reb A, a natural low‐calorie sweetener, has anti‐lipid peroxidation, hypolipidemic and antioxidant properties. Reb A (200 mg/kg) treatment could exert a protective effect against diabetic lipid oxidation in STZ‐induced diabetic rats, by promoting the activity of enzymatic antioxidants (Saravanan and Ramachandran 2013). A recent study suggested that Reb A attenuated aging by reducing cellular reactive oxygen species (ROS) levels in response to oxidative stress and the ectopic accumulation of neutral lipids (Li, Wang, et al. 2021; Li, Zhu, et al. 2021).

### 3.4 Anti‐Tumor

As illustrated in Table 3, numerous studies have confirmed that STV demonstrated anticancer activity against a broad variety of cancers, including bladder cancer, breast adenoma, colon cancer, and several human tumor cell lines (Chen et al. 2022; Guo et al. 2024; Hagiwara et al. 1984; Khare and Chandra 2019; López et al. 2016; Toyoda et al. 1997; Ukiya et al. 2013; Ren et al. 2017; Velesiotis, Kanellakis, and Vynios 2022). The most recent has uncovered that STV effectively inhibited hepatocellular carcinoma (HepG2 and Huh7 cells) both in vitro and in vivo by modulation of NF‐κB and PI3K/Akt signaling (Guo et al. 2024). The different groups have also verified that stevioside and Reb A exhibited antiproliferative and antimigratory on breast cancer cell line ERα^−^/ERβ^+^MDA‐MB‐231 cells and SK‐BR‐3, while promoting migration and adhesion of ERα^+^ MCF‐7 cell lines (Velesiotis, Kanellakis, and Vynios 2022; Khare and Chandra 2019). Furthermore, prior reports have indicated that long‐term consumption of STV did not promote bladder carcinogenesis in N‐butyl‐N‐(4‐hydroxybutyl)‐nitrosamine (BBN) induced rats (Hagiwara et al. 1984). Recent studies have found that stevia glycosides can induce apoptosis by activating GSK‐3β and endoplasmic reticulum (ER) stress signaling pathways, thereby inhibiting bladder cancer development (Chen et al. 2022).

**TABLE 3:** The advancements in the anti‐tumor activity of stevia glycosides were observed in vitro.

Tumor	Tumor type	Intervention dose	Intervention time	Intervention outcome	Reference
Hepatocellular carcinoma	HepG2	1.2–4.1 μM stevioside	1, 3, and 5 days	Inhibit HCC cells in vitro and in vivo by the reduction of pro‐inflammatory SASP components mediated via NF‐κB and PI3K/Akt signaling	Guo et al. (2024)
	Huh7 cells				
Breast cancer	MCF‐7 cells	2, 5, 10, and 20 μM Reb A (Reb A: 64.7%, Stevioside: 19.3%), for MCF‐7 cells; 100, 250, and 400 μM Reb A for MDA‐MB‐231 cells	24 and 48 h	Promoted MCF‐7 cells migration and adhesion	Velesiotis, Kanellakis, and Vynios (2022)
	MDA‐MB‐231 cells			Reduced MDA‐MB‐231 cells migratory and metastatic potential	
	MDA‐MB‐231 cell line	5, 10, 25, 50, 75, and 100 μM stevioside	24 h/48 h	Inhibit the proliferation and promote apoptosis	Khare and Chandra (2019)
	SKBR3 cell lines				
	SK‐BR‐3 cell lines	1.2–4.1 μM stevioside	3 h	Enhance the toxicity of cancer cells and induce their apoptosis	López et al. (2016)
	Breast adenoma	5% stevioside	104 weeks	No significant change	Toyoda et al. (1997)
Bladder cancer	T24 cell lines	2, 5, and 10 mg/kg of stevioside	24 and 48 h	Inhibited the proliferation of bladder cancer cells and induced their intrinsic apoptosis sparing normal cells by activation of GSK‐3β and ER stress signaling pathways	Chen et al. (2022)
	5637 cell lines				
	Bladder cancer	5% stevioside	32 weeks	No significant change	Hagiwara et al. (1984)
Colon cancer	HCT116 cell line	1.0, 1.5, 2.0, and 2.5 μg/mL stevioside	72 h	Show antiproliferative effects through inhibition of cyclin‐dependent kinases	López et al. (2016)
	HT‐29 cell line	0.5, 1, 2.5, and 5 μM stevioside	24, 48, and 72 h	MAPK and ROS‐mediated apoptotic cell death	Ren et al. (2017)
Cervical cancer	HeLa cell line	1–250 μg/mL *Stevia rebaudiana* ethanolic extract	72 h	Anti‐proliferation by inhibition of cyclin‐dependent kinases	López et al. (2016)
Pancreatic cancer	MiaPaca‐2 cell line				
Leukemia	HL60 cell line	1.2–4.1 μM stevioside	3 h	Enhance the toxicity of cancer cells and induce their apoptosis	
Lung cancer	A549 cell line				Ukiya et al. (2013)
Gastric cancer	AZ521 cell line				

Abbreviations: ER, endoplasmic reticulum; GSK‐3β, glycogen synthase kinase 3; HCC, hepatocellular carcinoma; MAPK, mitogen‐activated protein kinase; NF‐κB, nuclear factor kappa‐B; PI3K/Akt, phosphoinositide‐3‐kinase/serine/threonine kinase B; ROS, reactive oxygen species; SASP, senescence‐associated secretory phenotype.

Oral administration of 5% STV with food in F344 rats for 104 weeks significantly reduced the incidence of breast adenoma in female rats and mitigated the severity of chronic kidney disease in male rats, without notable alterations in the development of neoplastic or non‐neoplastic lesions (Toyoda et al. 1997). Recent studies have revealed that stevia glycosides not only effectively inhibited the proliferation and promoted apoptosis of human breast cancer cell lines MDA‐MB‐231 and SKBR3 but also enhanced the sensitivity of breast cancer to chemotherapeutic agents (Khare and Chandra 2019). An investigation of the tumor proliferation inhibition effects of 37 steviol derivatives confirmed that the majority of these derivatives exhibit significant inhibitory effects on the proliferation of leukemia (HL60), lung cancer (A549), gastric cancer (AZ521), and breast cancer (SK‐BR‐3) cell lines (Ukiya et al. 2013). Subsequent studies demonstrated that the free radical scavenging activity and cell proliferation inhibition sensitivity of stevia leaf ethanol extract (SREE) on cancer cells of cervical cancer (HeLa), pancreatic cancer (MiaPaca‐2), and colon cancer (HCT116) followed the order: HeLa > HCT116 > MiaPaca‐2 (López et al. 2016). In addition, stevia glycosides have been shown to induce cytotoxicity in colon cancer through apoptosis mediated by reactive oxygen species and mitogen‐activated protein kinase signaling pathways (Ren et al. 2017).

The aforementioned studies offer compelling evidence for the inhibitory impact of stevia and its derivatives on various types of cancer. However, the precise mechanisms through which they exert their effects on different tumors still necessitate further investigation. Additionally, the clinical therapeutic potential of stevia and its derivatives for treating tumors has yet to be fully verified.

### 3.5 Enhance Cardiovascular System Function

Cardiovascular disease (CVD) is characteristic by the dysfunctions of the heart, arteries, and veins, and its increasing incidence has seriously endangered health around the world. Lifestyle changes, lowering cholesterol, blood sugar levels, and weight are effective ways to prevent CVD. Stevia glycosides as natural sweeteners have been shown through physiological and pharmacological studies to exhibit various pharmacological activities, including maintaining blood lipid levels, promoting blood coagulation, and enhancing vascular function (Hussein et al. 2020; Maki et al. 2008; Raghavan et al. 2023; Ragone et al. 2017; Ren et al. 2017).

#### 3.5.1 Cardioprotective Effect

STV and Reb A, as stevia leaf medicinal ingredients, have beneficial effects on enhancing human heart health. These included reducing blood pressure, regulating heartbeat, relaxing blood vessels, inhibiting vasoconstriction, and diuresis (Ilias, Hamzah, et al. 2021). Furthermore, STV has been shown to activate the PI3K/AKT signaling pathway by upregulating PPAR‐γ, which can attenuate neuronal apoptosis and inflammation induced by middle cerebral artery occlusion/reperfusion (MCAO/r). This suggests that STV may play a protective role in neuronal damage caused by cerebral blood deficiency reperfusion (Gardana, Scaglianti, and Simonetti 2010; Zhang 2021). Additionally, STV has been found to ameliorate heart injury by reducing myocardial oxidative stress and maintaining Ca^2+^ homeostasis through myocardial NF‐κB/TGF‐β1/Smad (drosophila mothers against decapentaplegic protein) signaling pathway in isoprenaline‐induced myocardial fibrosis and myocardial hypertrophy (Ragone et al. 2017; Wang, Shen, et al. 2019).

#### 3.5.2 Prevention of Atherosclerosis

Diterpene glycosides derived from stevia glycosides, such as stevia glycoside and Reb A, have been evaluated for their efficacy in lowering cholesterol levels. These glycosides are potential candidates for the treatment and prevention of atherosclerosis caused by circulating lipid retention in the arterial subendothelial layer (Chen et al. 2019). STV can activate insulin signaling, improve the antioxidant effect of adipose tissue and vascular wall, and inhibit the formation of atherosclerosis in insulin‐resistant obese mice (Geeraert et al. 2010; Ilias, Hamzah, et al. 2021). An investigation aimed at assessing the anti‐atherosclerotic activities of eleven labdane diterpenoid stevelins isolated from *S. rebaudiana* revealed that most compounds significantly prevented the formation of macrophage foam cells induced by oxidized LDL (ox‐LDL) (Cheng et al. 2023). This finding indicated that these stevelins hold potential promise as candidates for the treatment of atherosclerosis.

#### 3.5.3 Lower Blood Pressure

Inhibition of angiotensin‐converting enzyme (AGE) activity has been one of the main strategies for treating hypertension. The inhibition of AGE activity was 26.60%, 59.56%, and 74.38% by the ethanolic extract of Stevia leaf, Stevia leaf proteolysis, and stevia glycoside (purity 95%), respectively. Their inhibitory effect on AGE is dose‐dependent, and appropriate doses can avoid hypertension or hypotension (Wang, Wu, et al. 2019). Isosteviol, the hydrolysis product of STV under acidic conditions, also has benefits in lowering blood pressure. It has been reported that intravenous stevia glycoside treatment can reduce the blood pressure of rats in a dose‐dependent manner (Melis 1992). Another study, which evaluated the impact of stevia glycoside on blood pressure, confirmed that intravenous administration of stevia glycoside exhibits a notable antihypertensive effect by inhibiting calcium influx in spontaneously hypertensive rats (SHRs) (Lee et al. 2001). However, oral supplementation with Reb A (0.025 g/kg BW/day) for 8 weeks did not affect blood pressure or blood glucose in male Goto‐Kakizaki (GK) rats (Dyrskog et al. 2005). The primary reasons for different research conclusions may include variations in animal models, different drug administration routes, and the purity of the drugs.

Clinical studies have demonstrated that administering stevia glycoside (15.0 mg/kg/day) before lunch and dinner for six consecutive weeks significantly lowered blood pressure in patients with mild essential hypertension. However, its antihypertensive effect was not significantly different from that of the placebo group, suggesting that oral ingestion of crude stevia glycoside is safe in Brazil (Ferri et al. 2006). In another study, Chinese patients with mild essential hypertension consumed capsules or placebo containing 250 or 500 mg of stevia glycoside powder three times daily for one to two years (Chan et al. 2000). The results indicated that the mean systolic blood pressure (SBP) and diastolic blood pressure (DBP) in the stevia glycoside group were significantly reduced compared to baseline. The antihypertensive effect was sustained over time, and no adverse side effects were observed (Hsieh et al. 2003).

### 3.6 Improved Kidney Function

The content of p‐aminohippurate acid (PAH) can be used to measure effective renal plasma flow (ERPF) and the function of the renal excretory system. Early studies have shown that intravenous infusion of STV accelerated the elimination of PAH, increased fractional sodium excretion, urinary flow as a percent of glomerular filtration rate, and glucose clearance in Wistar rats (Melis 1992). Crude extract of *S. rebaudiana* promoted renal water and sodium excretion in male Wistar rats (Melis 1999). Investigation on the impact of STV on the transepithelial transport of PAH in rabbit proximal renal tubules revealed that it has no harmful effect on tubular function at a pharmacological concentration of 0.70 mM (Jutabha, Toskulkao, and Chatsudthipong 2000). Stevia residue extract also has the potential to attenuate hyperuricemia and renal protective effects by downregulated inflammatory response (Mehmood et al. 2020). Subsequent study supported that stevia residue extract protected against uric acid‐associated renal injury by inhibition of inhibiting nuclear factor kappa‐B/NLR Family, pyrin domain‐containing protein 3 (NF‐κB/NLRP3) in mice (Mehmood et al. 2022; Hsu et al. 2002).

Numerous studies have indicated that stevia glycosides and their metabolites exhibit favorable ameliorative effects on various forms of kidney damage, encompassing renal cysts, acute kidney injury, and renal fibrosis (Altamimi et al. 2023; Kaur et al. 2021; Rotimi et al. 2018; Shen et al. 2020). Specifically, STV has been shown to inhibit oxidative damage in the liver and kidney of high‐fat/low‐streptozotocin diabetic rats by preventing DNA fragmentation (Rotimi et al. 2018). Furthermore, STV has been found to alleviate renal fibrosis in rats with unilateral ureteral obstruction (UUO) through the activation of PPARγ, which subsequently downregulates inflammatory signaling pathways (Shen et al. 2020). Additionally, STV attenuated acute kidney injury via activating of PPAR‐γ in rhabdomyolysis‐induced rats (Kaur et al. 2021). Recently, it reported that STV alleviated renal tubular epithelial cell injury and renal histological damage by inhibiting the gasdermin D pathway both in cisplatin‐ and ischemia/reperfusion‐induced AKI mouse models (Qiao et al. 2024). Steviol, a major metabolite of stevia glycoside, can slow the growth of renal cysts by decreasing the expression of aquaporin 2 (AQP2) or cystic fibrosis transmembrane conductance regulator (CFTR) in polycystic kidney disease (PKD) (Noitem et al. 2018). Isosteviol sodium salt (STVNa), a diterpenoid compound derived from STV hydrolysis, slowed renal cyst progression by suppressed CFTR and mammalian target of rapamycin/ribosomal protein S6 kinase (mTOR/S6K) expression, stimulated AMP‐activated protein kinase (AMPK) in autosomal dominant PKD (ADPKD) mouse model (Yuajit et al. 2014).

Although the renal protective effects of stevioside and its metabolite have been verified in various animal models, its pharmacological efficacy requires more comprehensive clinical studies for corroboration. Additionally, deeper research is required to explore the target of STV.

### 3.7 Antibacterial

The antimicrobial activity exhibited by *S. rebaudiana Bertoni* leaf extracts (STE) against various microorganisms has garnered considerable attention. Numerous studies have consistently demonstrated that STE possesses bacteriostatic effects on a range of microorganisms, including *Lactobacillus reuteri*, *Escherichia coli* , *Staphylococcus aureus* , *Bacillus subtilis* , and *Candida albicans* (Amaro‐Luis, Adrián, and Díaz 1997; Deniņa et al. 2014; Gamboa and Chaves 2012; Pandiyan et al. 2023). The latest report found that STE shows good antibacterial activity on antibiotic‐resistant *E. coli* by disrupting the permeability of the cell wall and the membrane (Chen, Yi, et al. 2024; Chen, Li, et al. 2024).

There has been increasing research on the utilization of green nanoscience to improve the bioactive activity and reduce the cytotoxicity of STE (Pandiyan et al. 2023; Srihasam et al. 2020; Alshawwa et al. 2022; Abdelhai et al. 2024). In these studies, noble metal nanoparticles (NPs) such as silver (Ag) and copper (Cu), as well as metal oxide NPs including nickel oxide (NiO) and iron (III) oxide (Fe_2_O_3_), were employed in the preparation of *S. rebaudiana* ‐mediated NPs. NiO nanoparticles prepared using Stevia leaf extract demonstrated a significantly stronger inhibitory effect against Gram‐negative bacteria ( *E. coli* ) compared to Gram‐positive bacteria (*B. subtilis and Streptococcus pneumonia*) and fungi (*fungi Aspergillus niger* and *Aspergillus funigatus*). This enhanced inhibition is attributed to the absence of a peptidoglycan layer in the outer membrane of Gram‐negative bacteria (Srihasam et al. 2020). Consistent with this, STE‐nanoscale alpha hematite (α‐Fe_2_O_3_) NPs inhibited *E. coli* and *S. aureus* bacterial growth in a dose‐dependent manner (Alshawwa et al. 2022). Copper nanoparticles and silver Nanoparticles biosynthesis by STE both exhibited a broad‐spectrum impact with bactericidal and fungicidal effects. In short, STE‐nanoparticles offer a promising alternative to commercial antibiotics in addressing the challenge posed by multidrug‐resistant (MDR) microorganisms, but the antimicrobial mechanism of STE needs further study.

## 4 Toxicological Effects and Safety

No evidence suggests that stevia can cause DNA damage, potential carcinogenicity, reproductive teratogenicity, or embryotoxicity. Studies have shown that doses up to 2000 mg/kg body weight of STV do not induce DNA strand breakage or chromosome damage in rats and mice, and STV coarse crystals have been verified as non‐mutagenic (Saeidnia and Abdollahi 2013). Both the 2005 Joint Expert Committee on Food Additives and international food safety agencies have concluded that stevia, STV, and Reb A can be used widely without concerns about genotoxicity (Abo Elnaga et al. 2016; Liu, Pan, and Wu 2018). In 2023, the EFSA Panel on Food Additives and Flavorings (FAF Panel) determined that an acceptable daily intake (ADI) of 4 mg/kg body weight per day, expressed as steviol equivalents, is suitable for the proposed food additive.

Bioactive compounds of stevia, including chlorophyll, lutein, STV, and Reb A, exhibit notable anti‐inflammatory and anti‐cancer properties (Salvador, Sotelo, and Paucar 2014). In 1999, the Food and Agriculture Organization and World Health Organization (FAO/WHO) Expert Committee on Food Additives unequivocally stated that STV poses no risk of carcinogenicity (Gupta et al. 2016). Furthermore, a recent study found that consuming a beverage sweetened with 25% of the ADI of stevia for 4 weeks had no significant impact on the human gut microbiome, fecal short‐chain fatty acid levels, or fasting cardiometabolic measures, compared to beverages sweetened with 30 g of sucrose (Kwok et al. 2024).

The Matogloso Indian tribes in Paraguay use stevia leaves to make tea or as an oral contraceptive drink, and to date, no teratogenicity or embryotoxicity induced by STV in the reproductive system has been reported by national or international regulatory bodies (Gupta et al. 2016). Several studies have found that pure STV and Reb A have no adverse effects on the male or female reproductive system (Usami et al. 1995; Koubaa et al. 2015), On the contrary, Reb A manifested deleterious effects on reproductive indices in aged mice including increase of abnormal estrous cycles and the number of corpora lutea (Ngekure et al. 2019). Subsequent studies demonstrated that high‐purity and high doses of Reb A were safe for reproduction, and testicular histopathology with 28‐ and 90‐day tests in the males and females (Gupta et al. 2016). These findings suggest that while sweeteners may have adverse effects on the reproductive system of the elderly, the specific circumstances and conditions under which these effects occur need further investigation.

The metabolic safety of Reb D and Reb M has been well‐established through various studies (Toyoda et al. 1997; Nikiforov et al. 2013; Purkayastha et al. 2014). As in the earlier study, Reb D had no significant impact on the daily life and behavior of Sprague Dawley (SD) rats, and no toxicological effects were observed after blood, serum, or urine analysis (Toyoda et al. 1997). The hydrolysis of Reb A, Rebaudioside B, Reb D, Reb M, and stevia was investigated using the fecal homogenate of healthy volunteers under anaerobic conditions in vitro. The results indicated that most of the stevia glycosides were hydrolyzed to steviol within 8 h of incubation, and the entire metabolic process was completed within 24 h. The hydrolysis time of high‐concentration STV was slightly longer than that of low concentration, but the concentration of STV had no significant effect on metabolic amount. Additionally, the metabolic rate and degree of hydrolysis were not significantly different among the various STV (Nikiforov et al. 2013). Genetic testing further confirmed the safety of Reb M, with an acute oral maximum tolerated dose (MTD) value greater than 10,000 mg/kg·bw in both male and female mice, which is equivalent to 1312 times the estimated maximum intake. This finding indicated no genotoxicity associated with Reb M (Purkayastha et al. 2014).

## 5 Acceptable Daily Intake

The intake of stevia is closely related to its relative molecular weight, with the stevia equivalent being converted to 0.33 times the weight of Reb A and 0.4 times the weight of STV. According to the FAO/WHO Joint Expert Committee on Food Additives, the ADI for stevia, expressed as steviol equivalent, is 0–2 mg/kg body weight per day. Specifically, the ADI for Reb A is 0–12 mg/kg body weight per day, and for STV, it is 0–4 mg/kg body weight per day (Gupta et al. 2016). The U.S. Food and Drug Administration (FDA) has approved stevia glycosides (SGs), with STV and Reb A as the main ingredients, as safe dietary supplements. The ADI for SGs in humans is set at 7.9 mg/kg body weight per day, and for rats, it is 25 mg/kg body weight per day (Wu, Zhao, and Shi 2021). Furthermore, the Dietary Supplement Health and Education Act (DSHEA), passed in 1994, permits the use of SGs as an ingredient in dietary supplements.

## 6 Other Applications

### 6.1 Applications in the Food Industry

Rebaudioside A is widely utilized as a sweetener in functional foods and snacks. When combined with low‐energy functional sweeteners such as erythritol and isomaltose oligosaccharide, Reb A serves as the primary sweetener in the development of functional sugar‐free yogurt tailored for diabetes and obesity patients (Yang &Hu 2013). This innovation provides a fresh impetus for the promotion of functional yogurt (Geuns et al. 2007). Building upon traditional cake production techniques, cakes have also been processed using Reb A mixed with other sweeteners in place of sucrose. These cakes offer dual benefits of nutrition and health care, making them highly marketable (Yang et al. 2012). Furthermore, it is feasible to produce jelly, frozen drinks, preserved fruit, and numerous other foods by utilizing stevia with a high Reb A content as a sucrose substitute. The aftertastes of Reb D and Reb M ice creams are much sweeter, more pleasant, creamier, and milkier than Reb A ice creams, which is the most widely used glycoside in the food industry (Muenprasitivej et al. 2022). In Brazil, steviol glycosides are the most found high‐intensity sweeteners (HIS) in biscuits, and consuming bakery products containing steviol glycosides poses no toxicological risks (Nicoluci et al. 2024).

### 6.2 Applications in Medicine

Stevia glycosides are added to some medicines as sucrose substitutes. For instance, syrups often contain a high sugar content, typically up to 65%, which can affect the efficacy of the drug and harm the human body. The use of STV to replace sucrose in traditional Chinese medicine preparations has been proposed (Qian 2005). Sucrose in some oral liquids has greatly limited the use of drugs for diabetics. To address this limitation, stevia is now added to some oral liquids as a sucrose substitute, such as in an inosine oral solution where steviol glycoside serves as a flavoring agent (Ruan 1994; Qian 2005). The use of steviol glycosides to replace sucrose in the production of sugar‐free medications has been applied to sugar‐free Yiqi Buxue granules (Wang, Tang, and Zhong 2011).

### 6.3 Crop Storage Applications

It has been reported that incorporating 0.5% of stevia polysaccharide from green feed could enhance the silage quality of whole corn forage bean, wolftail, and other forage crops (Xie 2023a, 2023b). Research investigating the antibacterial efficacy of *Equisetum arvense* and *S. rebaudiana* extracts against the growth and mycotoxin production by *Aspergillus flavus* and *Fusarium verticillioides* in maize seeds revealed that the extract of *Equisetum* and a mixture 1:1 of *Equisetum–Stevia* has shown that the extract of *Equisetum* alone, as well as a 1:1 mixture of *Equisetum* and *Stevia*, may effectively inhibit both the growth of aflatoxin‐producing fungi and aflatoxin production at elevated water activity levels (Garcia et al. 2012), but its mechanism is still unclear. Investigation for the effects of silver Nanoparticles biosynthesis by STE (STE‐AgNPs) on germination, growth, and biochemical parameters of three agricultural crop seeds (rice, maize, and peanut) have demonstrated that the impact of STE‐AgNPs betaine on crops depends on its size, the photodamage is higher in relatively smaller size STE‐AgNPs treated maize, and it also shows strong inhibitory action against *A. niger fungi* isolated from ground nut seeds (Prasad et al. 2017). Therefore, STE could be applied as an alternative treatment to control aflatoxigenic mycobiota in maize.

### 6.4 Applications in Agriculture

Recent studies have revealed that steviol glycosides effectively enhance the performance of livestock and poultry, boost feed utilization, and regulate the gastrointestinal microbiota. Research has unequivocally demonstrated that the incorporation of STV/Reb A into the diet of weaned piglets significantly enhances the composition and relative abundance of their gut microbiota, thereby promoting improved growth performance. (Wang, Shi, et al. 2014). Supplementing goat diets with STV at concentrations ranging from 400 to 800 mg/kg forage (approximately 270–541 mg/kg of the total diet) resulted in an increase in dry matter intake of both forage and the overall diet, indicating that STV holds promising potential as a feed additive for enhancing feed intake (Han et al. 2019). Dietary supplementation with STE (STV or another specified compound) could enhance the laying performance and eggshell quality of aged breeder hens by increasing estrogen synthesis, calcium levels, and antioxidant capacity within their reproductive organs (Jiang et al. 2020). STV, at concentrations of 217.68 and 215.21 mg/kg, enhanced growth performance, liver antioxidant capacity, and immune function in juvenile *songpu mirror carp* ( *Cyprinus carpio* ) (Wang et al. 2021). Recently, steviol glycosides have demonstrated the capacity to enhance growth performance, optimize rumen fermentation processes, and foster microbial diversity among Hu sheep following a 90‐day feeding period (Zhang, Li, et al. 2024; Zhang, Chen, et al. 2024). Furthermore, dietary supplementation with steviol glycosides in weaned calves has shown favorable impacts on rumen development, achieved through the regulation of the rumen bacterial community (Wang et al. 2024). The above research indicated that adding STV to livestock feed during the feeding process contributes to their growth and the maintenance of intestinal microecology.

## 7 Conclusion

Although the safety of stevia glycosides remains a subject of debate, recent evidence overwhelmingly supports their reliability. Stevia glycosides are low‐calorie, natural, non‐toxic, and possess numerous functional properties, making them versatile in the sweetener market. They exhibit promising pharmacological activities in areas such as hypotension, hypoglycemia, anti‐diabetes, anti‐inflammatory, antibacterial, and anti‐tumor treatments, although the underlying mechanisms require further investigation. Stevia glycosides have diverse applications in the food industry, medicine, animal husbandry, and crop production. However, the stevia market in China is currently in its ascending phase, leading to overcapacity. Research on stevia primarily focuses on the upstream production process, while downstream application research is still in its nascent stages. Therefore, it is imperative to deepen our understanding of stevia, grasp the sweetness characteristics of its monomers, enhance the quality of stevia products, expand the domestic market, unleash its potential, foster the development of the stevia industry, and ultimately improve economic and social benefits.

## Author Contributions

**Aoyi Wang:** investigation (lead), writing – original draft (equal), writing – review and editing (equal). **Huiqin Hu:** formal analysis (equal), visualization (equal), writing – review and editing (equal). **Yadan Yuan:** formal analysis (equal), investigation (equal), visualization (equal), writing – review and editing (equal). **Shiran Mei:** formal analysis (equal), investigation (equal), writing – review and editing (equal). **Guoxue Zhu:** formal analysis (equal), visualization (equal), writing – review and editing (equal). **Qiaoyan Yue:** writing – review and editing (equal). **Yanliang Zhang:** conceptualization (equal), writing – review and editing (equal). **Shujun Jiang:** conceptualization (lead), writing – original draft (lead), writing – review and editing (lead).

## Ethics Statement

This work does not involve animal or human studies for experimentation.

## Conflicts of Interest

The authors declare no conflicts of interest.

## Data Availability

No data were used for the research described in the article.
